# Filling the blanks in temporal intervals: the type of filling influences perceived duration and discrimination performance

**DOI:** 10.3389/fpsyg.2015.00114

**Published:** 2015-02-11

**Authors:** Ninja K. Horr, Massimiliano Di Luca

**Affiliations:** Centre for Computational Neuroscience and Cognitive Robotics, School of Psychology, University of BirminghamBirmingham, UK

**Keywords:** temporal perception, perceived duration, short-interval duration, duration distortions, filled-duration illusion, interval filling

## Abstract

In this work we investigate how judgments of perceived duration are influenced by the properties of the signals that define the intervals. Participants compared two auditory intervals that could be any combination of the following four types: intervals filled with continuous tones (*filled* intervals), intervals filled with regularly-timed short tones (*isochronous* intervals), intervals filled with irregularly-timed short tones (*anisochronous* intervals), and intervals demarcated by two short tones (*empty* intervals). Results indicate that the type of intervals to be compared affects discrimination performance and induces distortions in perceived duration. In particular, we find that duration judgments are most precise when comparing two isochronous and two continuous intervals, while the comparison of two anisochronous intervals leads to the worst performance. Moreover, we determined that the magnitude of the distortions in perceived duration (an effect akin to the filled duration illusion) is higher for tone sequences (no matter whether isochronous or anisochronous) than for continuous tones. Further analysis of how duration distortions depend on the type of filling suggests that distortions are not only due to the perceived duration of the two individual intervals, but they may also be due to the comparison of two different filling types.

## Introduction

Many factors other than the physical duration of an interval influence perceived duration (see Allan, 1979 for a classic and Grondin, 2010 for a recent overview). For example, perceived duration is influenced by the filling of the interval to be judged as highlighted by the well-known filled duration illusion, whereby filled intervals are perceived as longer than their empty counterparts. This effect has been observed in a wide range of experimental conditions, with the definition of “filling” varying across studies. Several studies used continuous signals as filled intervals (e.g., Goldfarb and Goldstone, 1963; Steiner, 1968; Craig, 1973; Wearden et al., 2007; Hasuo et al., 2014) and compared those to empty intervals, which are typically consisting solely of a short beginning and end marker or a gap in a continuous signal (see Wearden et al., 2007 for a comparison of those two variations). Another type of filled interval leading to the filled duration illusion is a sequence of short filler signals that is compared to an empty interval lacking such fillers (e.g., Buffardi, 1971; Thomas and Brown, 1974; Adams, 1977). The magnitude of the overestimation for the latter type of filled intervals has been shown to increase with the number of fillers (Buffardi, 1971; Schiffman and Bobko, 1977). This overestimation has been termed “Illusion of a Divided Time Interval” by ten Hoopen et al. (2008).

Duration judgments with filled intervals are mostly investigated with regularly-timed tones—that is, isochronous rhythms. However, it has recently been reported that the temporal structure of fillers influences perceived duration. For example, Matthews (2013) showed that isochronous intervals are perceived to last longer than accelerating or decelerating ones. Horr and Di Luca (2014) found that isochronous intervals are perceived to last longer than anisochronous ones and that this effect increases not only with the amount of anisochrony but also, like the filled duration illusion, with the number of fillers (this is in accordance with tendencies found in earlier studies, see Grimm, 1934; Thomas and Brown, 1974).

Overall, this line of research indicates that the type *and* structure of interval filling influences perceived duration. To gain further insight into the mechanisms underlying short interval duration perception also discrimination performance has to be investigated experimentally. Rammsayer and Lima (1991) reported that filled intervals made up of a continuous signal are discriminated better than empty intervals. It remains to be determined, whether this superior discrimination of filled as compared to empty intervals is only true for one type of filled intervals, namely intervals filled with a continuous signal (e.g., a continuous sound) or can as well be generalized over intervals filled with sequences of short filler signals (e.g., short tones). I further remains to be investigated how discrimination performance differs between such continuous and short filler intervals of different temporal structure.

In the present article we investigate how the type of interval filling affects perceived duration and discrimination performance using four types of auditory intervals: continuous, isochronous, anisochronous, and empty intervals. In Experiment 1 we investigate duration discrimination performance by having participants compare two intervals of the same type. In Experiment 2 we aim at quantifying the perceptual distortions for each interval type. To our knowledge, this is the first attempt to quantify how the type of filling influences the magnitude of the “filled duration illusion.” Such discrimination is important to understand the mechanisms involved in short-interval duration perception as it constraints the type of cognitive mechanisms employed in prospective time judgments.

## General methods

### Participants

A total of 35 healthy volunteers with normal auditory sensitivity participated in the experiments for course credits or a payment of 7 GBP/h. All participants were naive to the purpose of the study, reported normal auditory sensitivity and took part in only one of the experiments. The experimental data collection and storage followed the ethical guidelines of the Declaration of Helsinki and was approved by the Science, Technology, Engineering, and Mathematics Ethical Review Committee of the University of Birmingham.

### Experimental design

Participants performed a two-interval forced-choice task, deciding via button pressing which of two intervals had been the one of longer duration. A trial consisted of a 1000 ms standard interval and a comparison interval of 500, 700, 850, 1000, 1150, 1300, or 1500 ms duration spaced by a random interval between 2000 and 2300 ms. The order of standard and comparison intervals was random and counterbalanced across trials. Experimental stimuli constituting an interval were 1000 Hz 70 dB tones with 2.5 ms ramped onset and offset. Each interval consisted either of (a) a beginning and end tone lasting for 10 ms each (empty interval), (b) five 10 ms regularly-timed filler tones (isochronous interval), (c) five 10 ms irregularly-timed filler tones (anisochronous interval) or of (d) a tone lasting for the entire interval duration (continuous interval). For the anisochronous intervals, temporal irregularity was created by randomly moving the onset of individual filler tones inside a range of plus or minus half of the interstimulus interval (i.e., 250 ms in the standard interval). Stimuli were presented via headphones. Participants' individual response proportions were assessed in relation to the physical duration difference between interval types. The point of subjective equality (PSE) and the just noticeable difference (JND) were estimated using the Spearman-Kärber-Method as the first and second moment of the data obtained from each participant (Ulrich and Miller, 2004).

## Experiment 1: duration discrimination performance

To investigate differences in duration discrimination performance across interval types, we asked participants to compare two intervals of the same type (continuous, isochronous, anisochronous and empty).

### Materials and methods

Seventeen healthy volunteers (15 female, 21.7 ± 2.8 years) participated in Experiment 1. In each experimental trial, participants reported which of two intervals was longer. According to the different interval types, four conditions were defined: continuous, isochronous, anisochronous, and empty. Each of the four conditions was presented in a block. The sequences of blocks (conditions) were randomized for each participant. Every block contained eight repetitions of all seven possible durations of the comparison interval (Mayer et al., 2014). In every block the eight repetitions of each comparison duration were counterbalanced and pseudo-randomized according to which interval (standard or comparison) was presented first. In total participants made 224 duration comparisons in 4 blocks of 56 trials each. The entire experiment lasted about 40 min.

### Results

**Figure 1:**
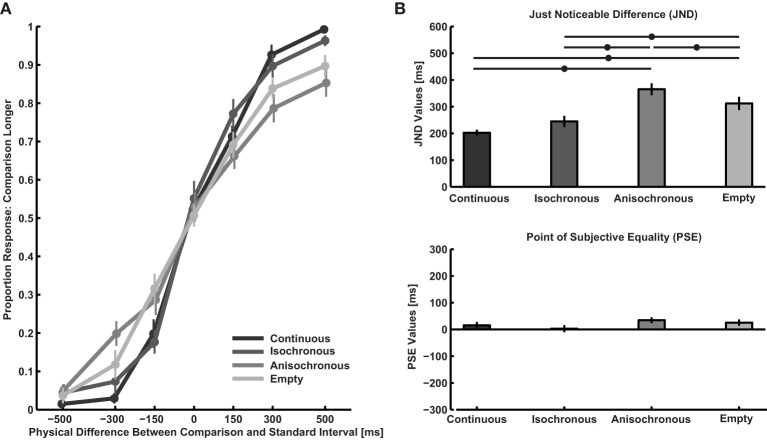
**Results of Experiment 1. (A)** Participants' responses recoded to indicate the proportion of responses where the comparison interval was judged longer than the standard interval as a function of physical duration difference. **(B)** Point of subjective equality (PSE) and just noticeable difference (JND) calculated from response proportions using the Spearman-Kärber method. Asterisks indicate differences in performance between intervals of different types as identified by the horizontal lines. Error bars are S.E.M.

In Figure 1A response proportions and Figure 1B PSE and JND values are displayed. Each participant's average JND is lower than 600 ms, which means that all of them were reasonably capable of performing the task. As participants were comparing two identical intervals, there should be no difference between PSE values across conditions [*F*_(3, 67)_ = 1.6, n.s]. More interestingly, there is a significant difference of JND values between conditions [*F*_(3, 67)_ = 15.4, *p* < 0.001, η^2^_*p*_ = 0.49].

*Post-hoc* tests indicate that the following differences are statistically significant: Duration discrimination is better for continuous than empty [paired sample *t*-test on JND, *t*_(16)_ = 3.9, *p* = 0.0013] and anisochronous intervals [*t*_(16)_ = 7.6, *p* < 0.001]. Discrimination is better for isochronous than empty [*t*_(16)_ = −2.2, *p* = 0.043] and anisochronous intervals [*t*_(16)_ = 4.5, *p* < 0.001]. Furthermore, discrimination is better for empty than anisochronous intervals [*t*_(16)_ = 2.4, *p* = 0.03]. There is no significant difference between continuous and isochronous intervals [*t*_(16)_ = 1.7, *p* = 0.12]. In short, continuous and isochronous intervals are discriminated best, followed by empty intervals, while discrimination performance is worst for anisochronous intervals.

## Experiment 2: distortions of perceived duration

To investigate whether distortions of perceived duration depend on the type of interval filling, we asked participants to compare perceived duration between all types of filled intervals and the empty intervals. Furthermore, we asked participants to compare the duration of different types of filled intervals.

### Material and methods

Eighteen healthy volunteers (12 female, 22.1 ± 3.3 years) participated in Experiment 2. In each trial, participants made their duration judgment for two intervals of different types. Six conditions were defined according to all possible combinations of the four interval types: (1) continuous/empty, (2) isochronous/empty, (3) anisochronous/empty, (4) continuous/isochronous, (5) continuous/anisochronous, and (6) isochronous/anisochronous. Each condition was presented in a separate block of trials. As in Experiment 1 sequences of blocks (conditions) and trials were fully randomized. The order of standard (1000 ms) and comparison (500–1500 ms) intervals was counterbalanced and the standard could be either of the two types of intervals presented in the block. Data from the combination of order and standard type is presented combined. Participants performed a total of 336 duration discrimination judgments resulting from 6 blocks of 56 trials each. The entire experiment lasted about 60 min.

### Results

**Figure 2:**
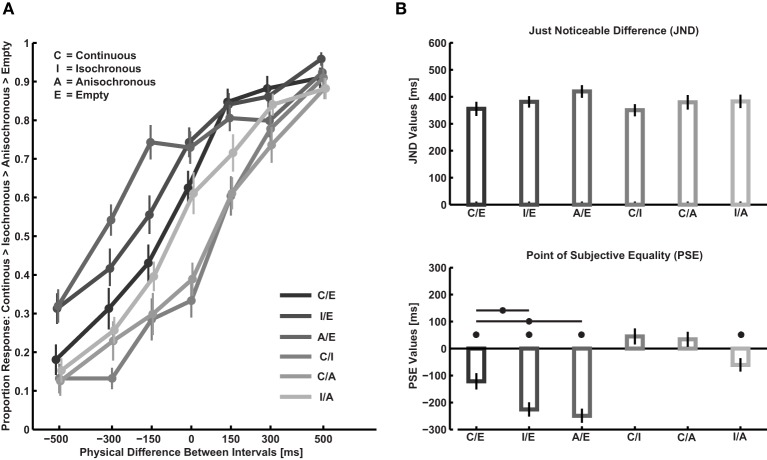
**Results of Experiment 2. (A)** Proportions of judging continuous > isochronous > anisochronous > empty as a function of duration difference between standard and comparison. **(B)** Point of subjective equality (PSE) and just noticeable difference (JND) calculated from response proportions using the Spearman-Kärber method. Asterisks indicate a significant difference of the PSE from zero and between the three conditions comprising one empty interval. Error bars are S.E.M.

Figure 2A shows response proportions and Figure 2B shows average PSE and JND values obtained across participants. Again as in Experiment 1, average JND values for each participant are lower than 600 ms indicating a reasonable performance. The PSE values depend on the type of filling [One-Way r.m. ANOVA: *F*_(5,107)_ = 23.4, *p* < 0.001, η^2^_*p*_ = 0.58]. In every conditions containing empty intervals PSEs are significantly lower than zero [single sample *t*-test on PSE against 0, continuous/empty: *t*_(17)_ = −4.0, *p* < 0.001; isochronous/empty: *t*_(17)_ = −8.6, *p* < 0.001; anisochronous/empty: *t*_(17)_ = −9.4, *p* < 0.001]. This indicates the presence of the filled duration illusion, that is, the duration of empty intervals being underestimated as compared to filled intervals. Isochronous intervals are perceived as longer than anisochronous ones [*t*_(17)_ = −2.5, *p* = 0.025], whereas PSE does not differ from 0 when comparing continuous and isochronous [*t*_(17)_ = 1.5, *p* = 0.15] as well as continuous and anisochronous intervals [*t*_(17)_ = 1.2, *p* = 0.24]. The magnitude of bias (PSE value) is lower for continuous intervals than for isochronous intervals [paired sample *t*-test on PSE isochronous/empty vs. PSE continuous/empty: *t*_(17)_ = 3.0, *p* = 0.008] as well as for anisochronous intervals [PSE anisochronous/empty vs. PSE continuous/empty: *t*_(17)_ = 3.5, *p* = 0.003]. There is no significant difference between isochronous and anisochronous [PSE isochronous/empty vs. PSE anisochronous/empty *t*_(17)_ = 0.8, *p* = 0.43]. No significant difference is observed in JND values across conditions [One-Way ANOVA on JND, *F*_(5, 107)_ = 2.0, *p* = 0.09, η^2^_*p*_ = 0.10], with a tendency toward better performance in conditions where one of the compared stimuli is a continuous interval. A comparison of JND values between Experiment 1 and 2 indicates higher performance when comparing intervals of the same type rather than of different types [two sample *t*-test on average JND for each participant: *t*_(33)_ = 4.3, *p* < 0.001, 0.38 ± 0.02 ms vs. 0.28 ± 0.01 ms].

## General discussion

The present article investigates discrimination performance and perceived duration of four types of auditory intervals: continuous tones, isochronous sequences of tones, anisochronous sequences of tones, and empty intervals. Such interval types have been commonly used in experiments investigating the filled duration illusion and related distortions of perceived duration (e.g., Thomas and Brown, 1974; Rammsayer and Lima, 1991; Wearden et al., 2007), but until now they have never been systematically compared. We find that discrimination performance changes depending on the interval types to be compared. When comparing the same types of intervals, continuous and isochronous intervals are discriminated better than empty intervals. Discrimination performance for anisochronous intervals is worse than for all other interval types. The filled duration illusion is found to be stronger for tone sequences, both isochronous and anisochronous, than for continuous intervals. The result of the comparison of different types of filled intervals, however, indicates that there are no differences in duration judgments between continuous tones and tone sequences, and that isochronous sequences are perceived as longer than anisochronous ones.

### Discrimination performance

Differences in duration discrimination performance between interval types demonstrate that participants make use of the structure of interval filling to arrive at their duration estimates. That is, for the different interval types they use either different sources of information or there is a common mechanism that changes in precision depending on the interval types.

Our data indicates that when comparing intervals of the same type, continuous and isochronous intervals are better discriminated than empty ones. This is in line with the idea that higher sound energy in the interval improves discrimination performance (Carbotte and Kristofferson, 1973). However, empirical evidence that do not support this possibility (Creelmann, 1962; Abel, 1972). Rammsayer and Lima (1991) suggest that filled intervals are discriminated better than empty intervals because they elicit a higher neural firing rate, which is translated to a superior temporal resolution. This possibility would predict a better discrimination performance for sound sequences than for continuous intervals because a continuous sound would be subject to habituation (e.g., Polich, 1989). In addition, Horr and Di Luca (2014) hypothesized that due to neural entrainment (e.g., Lakatos et al., 2008; Cravo et al., 2013), stimuli in isochronous sequences should arrive at the point of highest neural responsiveness leading to further increase in neural response in isochronous intervals when compared to continuous intervals. However, our results (Figure 1B) do not show a significant difference between continuous intervals and isochronous sequences. Also the finding of anisochronous sequences being discriminated worse than continuous tones and empty intervals is not in accordance with a neural firing rate explanation. The higher temporal resolution caused by increased neural responses can therefore only account for the decrease in performance found with empty as compared to continuous and isochronous intervals, as the lack of difference between continuous and isochronous intervals and even more so the remarkably worse performance for anisochronous as compared to all other intervals remains unexplained.

Another possibility to explain the observed pattern of discrimination performance is to appeal to the number of cues available for a single duration judgment. It has been shown that filled intervals defined by auditory and visual stimuli provide redundant cues to duration that allow a statistically optimal increase in performance (Hartcher-O'Brien et al., 2014). Here we posit that in some conditions there are redundant cues related to duration also for unisensory stimuli and this could lead to better discrimination performance compared to the conditions where only one cue is available. In particular, Hartcher-O'Brien et al. (2014) identify the filling of the interval as an important factor that can modulate the modality of integration, as empty intervals consist of two markers that only allow the identification of two time points and of the subtended empty duration between them. In contrast, continuous tones allow duration estimates by using the overall sensed energy in addition to (and independently from) the information carried by the temporal difference between beginning and ending time points. For isochronous intervals, the regular temporal structure allows to estimate duration based solely on the interval between successive tones (if the number of tones is known). Although the same cue is present with anisochronous intervals, the random timing of tones should be actually deceptive and lead to a reduced precision in duration judgments. If we interpret our data along these lines, the pattern of results suggests that the base duration judgment performance is achieved with empty intervals. In filled intervals the brain can use additional duration cues if both intervals carry such cues, that is, with trials with two intervals of the same type as in our Experiment 1. Such cues can either increase (as in the case of isochronous intervals), but also decrease discrimination performance (as with anisochronous intervals). If two intervals of different types are compared, additional cues cannot be used, leading to a lower discrimination performance in all conditions in Experiment 2. Such cues are present while comparing anisochronous intervals but they decrease rather than increase discrimination performance. On the other hand, such cues cannot be compared directly with stimuli of different types, leading to lower discrimination performance in all conditions of Experiment 2.

### Distortions of perceived duration

The goal of Experiment 2 was to characterize duration distortions. PSE data shows that the effect of the filled duration illusion (e.g., Steiner, 1968; Buffardi, 1971; Thomas and Brown, 1974; Wearden et al., 2007; Hasuo et al., 2014) is present for every type of filled interval we tested. The data however indicates that the magnitude of the filled duration illusion is higher with isochronous and anisochronous than with continuous intervals. That is, PSE values are significantly lower for the comparison between isochronous/empty and anisochronous/empty than for continuous/empty intervals. We hypothesize that different additional duration cues present in filled intervals could be responsible for this. For example, for some comparison types participants could use neural response magnitudes, as there seems to be a positive relation between those and perceived duration (see Eagleman and Pariyadath, 2009). The difference in the results with continuous intervals and tone sequences could then be due to the comparatively lower neural response with continuous intervals due to neural adaptation (e.g., Polich, 1989). The higher peak of neural response with isochronous as compared to continuous intervals could further be due to neural entrainment, at the expected time points (Lakatos et al., 2008). Appealing to overall energy in neural responses is intriguing because it can account for the filled duration illusion, for the higher effect of tone sequences as compared to continuous tone and for the here replicated difference between isochronous and anisochronous intervals (Horr and Di Luca, 2014). An alternative explanation for the differentiation between isochronous and anisochronous intervals taken alone could be a logarithmic relationship between physical and perceived duration of intervals between tones (see Thomas and Brown, 1974; Matthews, 2013; Horr and Di Luca, 2014).

The attempt to account for the overall pattern of results in Experiment 2 by appealing to one of the discussed single mechanism is limited by two apparent internal inconsistencies of the data. (1) Even though the direct comparison of isochronous with anisochronous intervals leads to a noticeable difference in perceived duration, the magnitude of the filled duration illusion measured by comparing a filled to an empty interval is *not* different for isochronous as compared to anisochronous intervals. (2) Even though the direct comparison of tone sequences (both isochronous and anisochronous) with continuous intervals does not lead to a significant difference, the filled duration illusion is weaker for continuous sounds than for isochronous and anisochronous intervals (again measured by comparing a filled to an empty interval).

To investigate the magnitude of inconsistencies in our data, we used the PSE values from the different comparison conditions to calculate relative duration distortions for each interval type as described in Mayer et al. (2014). Here we can express PSE values as the difference in the two physical durations *PSE*_12_ = *D*_1_ − *D*_2_ that leads to identical perceived durations D′_1_ = D′_2_. As perceived duration can be expressed as *D*′ = *D* + $\overset{ˇ}{d}$, where $\overset{ˇ}{d}$ represents the distortions in perceived duration *D*′ from the objective duration *D*, we can formulate PSE as a function of perceived durations and distortions:

$$PSE_{12}=D_{1}-D_{2}={D^{\prime }}_{1}-\overset{ˇ}{d_{1}}-{D^{\prime }}_{2}+\overset{ˇ}{d_{2}}.$$

But because perceived durations *D*′_1_ and *D*′_2_ are identical at PSE, we can simplify the formula as the difference in duration distortion:

$$PSE_{12}=D_{1}-D_{2}=\overset{ˇ}{d_{2}}-\overset{ˇ}{d_{1}}.$$

In fact, PSE can be expressed not only relatively to the objective duration *D*, but also as the difference in duration distortion *d* from any value *a* as such:

$$PSE_{12}\;=\;\left(a\;+\;\overset{ˇ}{d_{2}}\right)\;-\;\left(a\;+\overset{ˇ}{d_{1}}\right)=\;\overset{ˇ}{d_{2}}-\overset{ˇ}{d_{1}}.$$

In the following, *d*_1_ and *d*_2_ will represent the relative distortion in perceived duration with respect to *a*, the average duration distortion in the experiment. If we want to express the six PSEs obtained in the conditions of Experiment 2, we can use the following system of equations:

$$\left[\begin{array}{l} pse_{continuous/empty}\\ pse_{isochronous/empty}\\ pse_{anisochronous/empty}\\ pse_{continuous/isochronous}\\ pse_{continuous/anisochronous}\\ pse_{isochronous/anisochronous} \end{array}\right]=\left[\begin{array}{cccc} -1 & 0 & 0 & 1\\ 0 & -1 & 0 & 1\\ 0 & 0 & -1 & 1\\ -1 & 1 & 0 & 0\\ -1 & 0 & 1 & 0\\ 0 & -1 & 1 & 0 \end{array}\right]\;\left[\begin{array}{l} d_{continuous}\\ d_{isochronous}\\ d_{anisochronous}\\ d_{empty} \end{array}\right],$$

that is:

p= M d.

If *d* were the absolute value of distortion, such system would have infinite solutions. But here we express *d* relatively to the average duration distortion in the experiment *a*, so that a single solution to this linear system can be approximated using the Moore-Penrose pseudoinverse *M*^+^:

$$d_{estimated}=M^{+}\;p\;.$$

**Figure 3:**
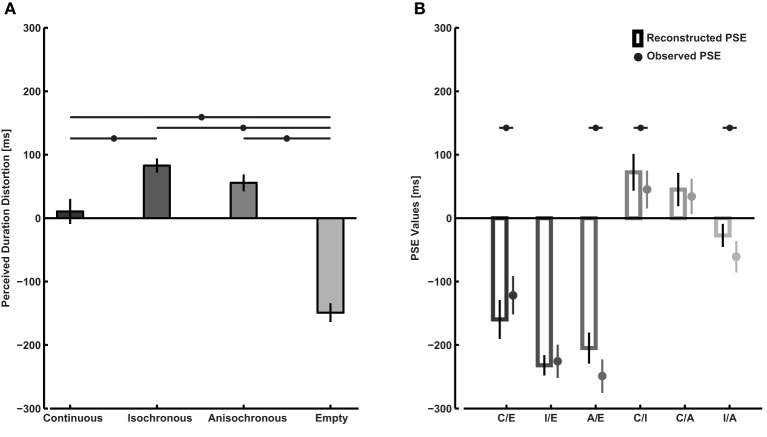
**Analysis of perceived duration distortions obtained from empirical PSE values. (A)** Perceived duration distortions relative to the mean of all intervals tested (the zero point on the vertical axis corresponds to the average distortion across the interval types tested) calculated from the empirical PSE values according to the system of equations described in the text. Asterisks represent a significant difference in distortion between two interval types as indicated by the horizontal lines. **(B)** Empirical PSE values compared to reconstructed PSE values from the calculated perceived duration distortions. Asterisks indicate a significant difference between the two, suggesting that factors other than duration distortion of the two intervals to be compared might have affected participant's judgments.

We apply this formula to the data obtained from each participant so to calculate the mean distortion in perceived duration for the four types of intervals tested (Figure 3A). Here, *d* = 0 refers to a duration distortion equal to the average duration distortion *a* over all interval types tested in Experiment 1 (see Mayer et al., 2014). Empty intervals are perceived as shorter than continuous intervals [paired sample *t*-test on d values, *t*_(17)_ = 5.2, *p* < 0.001], isochronous intervals [*t*_(17)_ = 14.5, *p* < 0.001], and anisochronous intervals [*t*_(17)_ = 8.4, *p* < 0.001]. Moreover, continuous intervals are perceived as shorter than isochronous ones [*t*_(17)_ = −2.5, *p* = 0.02]. There is no difference between continuous vs. anisochronous [*t*_(17)_ = −1.7, *p* = 0.10] nor isochronous vs. anisochronous [*t*_(17)_ = 1.5, *p* = 0.15] intervals. Reconstructing the PSE from calculated distortions is possible using:

$$p_{reconstructed}=\;M\;d_{estimated.}$$

Such formula makes it possible to determine whether PSE values in the comparison task were solely dependent on the sum of single interval distortions. The comparison between observed and reconstructed PSE values is displayed in Figure 3B. Observed and reconstructed data differ significantly as indicated by the interaction term of a Two-Way r.m. ANOVA on PSE values with factors condition and empirical/reconstructed [*F*_(5,85)_ = 5.3, *p* < 0.001, η^2^_*p*_ = 0.24]. The values for the continuous/empty [paired sample *t*-test on PSE, *t*_(17)_ = 2.8, *p* = 0.013], anisochronous/empty [*t*_(17)_ = −3.4, *p* = 0.003], continuous/isochronous [*t*_(17)_ = −2.7, *p* = 0.016], and isochronous/anisochronous conditions [*t*_(17)_ = −2.7, *p* = 0.015] differ significantly between empirical and reconstructed. Only the difference in the continuous/anisochronous [*t*_(17)_ = −0.9, *p* = 0.36] and isochronous/empty conditions [*t*_(17)_ = 0.47, *p* = 0.64] were not significant.

These inconsistencies indicate that distortions in two-interval forced-choice duration judgments do not solely depend on the perceived duration of the two intervals compared, which challenges the assumption of simple difference models (see e.g., Green and Swets, 1973; Thurston, 1994; Macmillan and Creelman, 2005). Context effects regarding the sequence in which stimuli are presented (e.g., Hellström, 1985, 2003; Dyjas and Ulrich, 2014) and the distribution of durations (e.g., Wearden and Ferrara, 1995; Brown et al., 2005; Wearden and Lejeune, 2008; Jazayeri and Shadlen, 2010) have frequently been reported in the literature. To test whether our results could be accounted for by hysteresis in duration judgments, i.e., if there is a distortion of perceived duration depending on the type of filling of the previous interval, we performed a 2 × 6 Two-Way r.m. ANOVA on PSE values with factors presentation order (which of the two intervals was presented first) and comparison type (the six comparison conditions, cf. Figure 2). In accordance with the literature (e.g., Hellström, 2003; Dyjas and Ulrich, 2014) we find a significant bias to judge the second interval as longer than the first one [*F*_(1, 17)_ = 12.7, *p* = 0.002, η^2^_*p*_ = 0.57] and as expected the factor comparison type is significant [*F*_(5, 85)_ = 23.45, *p* < 0.001, η^2^_*p*_ = 0.43]. Most importantly there is no significant interaction between the two factors order and comparison type [*F*_(5, 85)_ = 1.94, n.s.] suggesting that the inconsistencies in PSE we found cannot be accounted for by appealing to the presentation order of the intervals alone.

It thus remains unclear what are the factors inducing inconsistencies in the data across conditions, but one may speculate that different mechanisms could be used to compare durations when intervals to be compared are of the same type and of different type. We have discussed previously that duration judgments performed with the same type of intervals as in Experiment 1 could be aided by additional cues that are correlated to temporal duration (i.e., total energy and timing between successive tones). With the exception of isochronous and anisochronous intervals, the trials in Experiment 2 do not allow a direct comparison of additional cues to duration. Participants may have tried to map different cues to improve the comparison (i.e., mapping total energy in one interval to subinterval duration) thus creating response biases leading to one type of interval to be reported longer more often than the other (irrespectively of the physical duration). Such biases are dependent on the pair of stimuli involved in the comparison and could thus explain the inconsistencies we observed in our data.

## Conclusions

Our results highlight the influence of interval type on discrimination performance and perceived duration. The observed effects have several implications regarding the computational and neural mechanisms underlying duration judgments. Differences in discrimination performance can be explained by considering the presence of multiple cues for duration discrimination when comparing intervals of the same type. Also distortions in perceived duration can be accounted for by appealing to such additional cues, particularly neural response magnitude, which is higher for continuous and anisochronous stimuli compared to empty, but is even higher with isochronous stimuli due to neural entrainment. Interestingly, inconsistencies in the pattern of results indicate that duration judgments in a forced-choice comparison task are affected by factors other than distortions in perceived duration of the individual intervals. Such factors need to be taken into account to understand internal inconsistencies in duration comparisons between different interval types.

### Conflict of interest statement

The authors declare that the research was conducted in the absence of any commercial or financial relationships that could be construed as a potential conflict of interest.
